# Evaluating synergistic versus additive effects of the triplet regimen in metastatic castration-sensitive prostate cancer: a modeling analysis

**DOI:** 10.1093/jjco/hyaf211

**Published:** 2025-12-31

**Authors:** Shinro Hata, Shuntaro Suzuki, Hiroyuki Fujinami, Naoyuki Yamanaka, Toshitaka Shin

**Affiliations:** Department of Urology, Faculty of Medicine, Oita University, 1-1 Idaigaoka, Hasama-machi, Yufu, Oita 879-5593, Japan; Department of Urology, Faculty of Medicine, Oita University, 1-1 Idaigaoka, Hasama-machi, Yufu, Oita 879-5593, Japan; Department of Urology, Faculty of Medicine, Oita University, 1-1 Idaigaoka, Hasama-machi, Yufu, Oita 879-5593, Japan; Department of Urology, Faculty of Medicine, Oita University, 1-1 Idaigaoka, Hasama-machi, Yufu, Oita 879-5593, Japan; Department of Urology, Faculty of Medicine, Oita University, 1-1 Idaigaoka, Hasama-machi, Yufu, Oita 879-5593, Japan

**Keywords:** prostate cancer, darolutamide, docetaxel, drug synergy, independent drug action

## Abstract

The ARASENS trial demonstrated a significant overall survival (OS) benefit for a triplet regimen in metastatic castration-sensitive prostate cancer (mCSPC). We aimed to determine whether this benefit is synergistic or additive. Using a mathematical model of independent drug action and published data from the ARASENS and ARANOTE, we compared the observed OS of the triplet regimen to a predicted OS curve. Reconstructed individual patient data were compared using a Cox model. The observed OS was statistically superior to the predicted OS (hazard ratio [HR] 0.82, 95% CI 0.68–0.99; *P* = .047), indicating a clinical benefit ~18% greater than the expected additive effect. To address confounding by subsequent therapies, we analyzed time to initial subsequent anticancer therapy, which showed an even more pronounced greater-than-additive benefit (HR 0.57, 95% CI 0.44–0.74; *P* < .001). These findings suggest the triplet regimen provides an early therapeutic advantage that exceeds additive expectations, supporting an upfront combination strategy in mCSPC.

## Teaser

**Mini-abstract** A modeling analysis suggested the triplet regimen’s benefit in metastatic castration-sensitive prostate cancer is driven by a modest synergistic interaction, not just an additive effect of individual agents.

## Introduction

The therapeutic landscape for metastatic castration-sensitive prostate cancer (mCSPC) has evolved dramatically from androgen deprivation therapy (ADT) alone [1]. The addition of either docetaxel or an androgen receptor pathway inhibitor (ARPI) to ADT (doublet therapy) significantly improved overall survival (OS) and became the standard of care [2, 3]. Subsequently, several trials investigated triplet regimens by adding an ARPI to ADT and docetaxel. While the PEACE-1 and ARASENS trials demonstrated a survival benefit for abiraterone and darolutamide, respectively, the ENZAMET trial did not show a clear benefit for enzalutamide in this context, leaving the nature of the interaction between ARPIs and docetaxel a subject of debate [4–6]. The recent ARASENS trial established a new benchmark, demonstrating that adding darolutamide to standard treatment with docetaxel and ADT further reduced the risk of death compared to docetaxel and ADT alone [4].

While the clinical benefit is clear, the nature of the interaction between the added darolutamide and the docetaxel-based therapy is unknown. It is unclear whether the efficacy arises from a true synergistic interaction or simply the additive effect of two potent, independently acting therapies. This distinction is crucial for optimizing future combination strategies.

Therefore, the objective of this study was to quantitatively evaluate the potential for synergy in the ARASENS triplet regimen by applying a validated model of independent drug action to reported OS data.

## Materials and methods

This modeling study was based on publicly available data and was exempt from institutional review. Our primary endpoint was OS. To address potential confounding by subsequent therapies, we also performed a supplementary analysis using time to initial subsequent systemic anticancer therapy (TSSAT) as an endpoint. We digitally extracted Kaplan–Meier (KM) curves for both OS and TSSAT from the primary publications of the ARASENS (triplet vs docetaxel + ADT) [4] and ARANOTE (darolutamide + ADT vs ADT alone) [7] trials using WebPlotDigitizer (v5.2). At least 20 coordinate points were extracted from each curve. We applied a model of independent drug action to predict the outcome probabilities for both OS and TSSAT of the triplet regimen, *P*_predicted(*t*)_, under the assumption that its components act independently. This model calculates the expected outcome when drugs affect different patient subgroups or induce cell death through distinct, noninteracting mechanisms. The formula used was derived from the law of probabilistic independence:

*P*_predicted(*t*)_ = *P*_Dtx_ + ADT(*t*) × (*P*_daro_ + ADT(*t*)/*P*_ADT(*t*)_)

where *P*_Dtx_ + ADT(*t*) is the observed OS probability of the docetaxel + ADT arm in ARASENS, and the ratio represents the relative survival benefit of darolutamide derived from ARANOTE.

Individual patient data (IPD) were reconstructed from the observed and predicted KM curves using the “Shiny app for IPDfromKM,” which implements the IPDfromKM algorithm [8]. The extracted coordinate data were processed to ensure monotonic decrease using the MIN function in Microsoft Excel. A common time axis with 0.5-month intervals was established, and survival probabilities for each time point were calculated using linear interpolation with the FORECAST.LINEAR function.

For each endpoint (OS and TSSAT), the reconstructed IPD for the observed and predicted arms were compared using a Cox proportional hazards model to calculate the hazard ratio (HR) and 95% confidence interval (CI). The log-rank test was additionally used for the OS comparison. An HR < 1.0 with a CI not crossing 1.0 was considered indicative of synergy. All the statistical analyses were performed using EZR (Saitama Medical Center, Jichi Medical University, Saitama, Japan), a graphical user interface for R (The R Foundation for Statistical Computing, Vienna, Austria) [9]. A *P*-value < .05 was considered statistically significant.

## Results

The baseline characteristics of the patients from the ARASENS and ARANOTE trials are presented in Supplementary Table S1. The populations were broadly comparable in terms of key prognostic factors. The proportion of high-volume disease was 77.0% in the ARASENS population and 70.5% in the ARANOTE population. Similarly, the proportion of patients with high Gleason scores (≥8) was 78.2% in ARASENS and 68.3% in ARANOTE. However, a notable difference was observed in the proportion of patients with de novo metastatic disease, which was higher in the ARASENS trial (86.1%) compared to the ARANOTE trial (72.5%).

**Figure 1:**
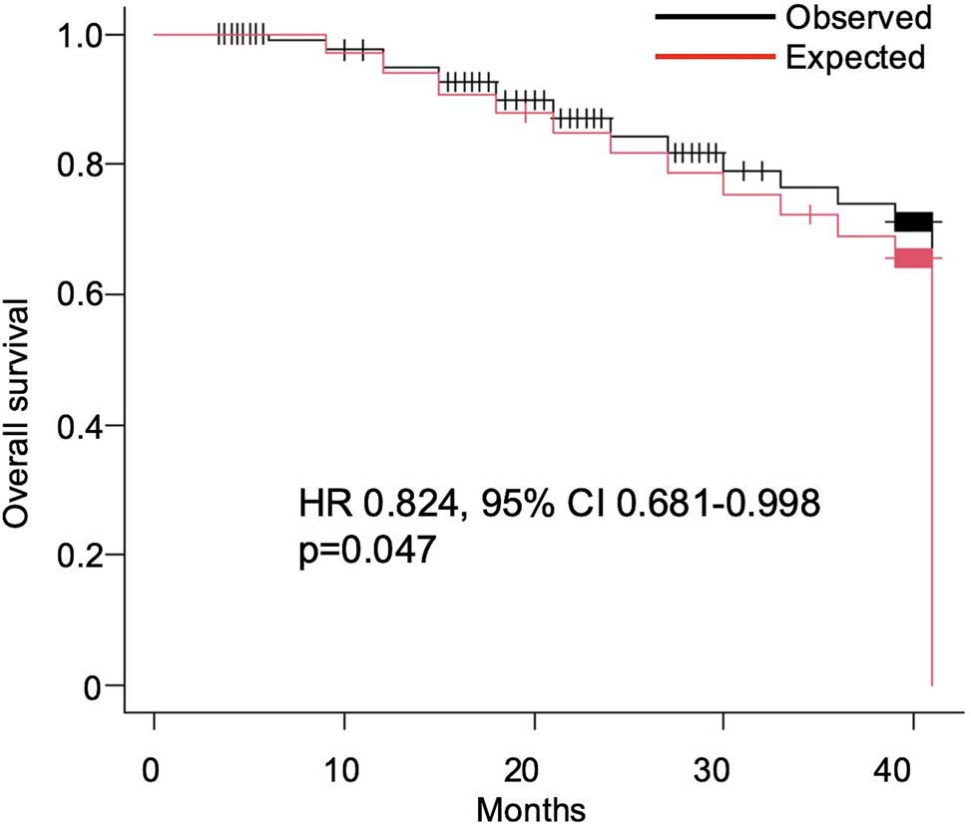
Kaplan–Meier curves of observed versus predicted overall survival. The black line represents the observed overall survival (OS) of the triplet regimen, based on reconstructed individual patient data (IPD) from the ARASENS trial. The red line represents the predicted OS, generated by an independent drug action model using data from both the ARASENS and ARANOTE trials. The hazard ratio (HR), 95% confidence interval (CI), and *P*-value shown were derived from a Cox proportional hazards model comparing the two reconstructed cohorts.

The KM curve for the observed OS of the triplet regimen was consistently superior to the predicted OS curve generated by the independent action model (Fig. 1). The log-rank test demonstrated a statistically significant difference between the two curves (*P* = .046).

**Figure 2:**
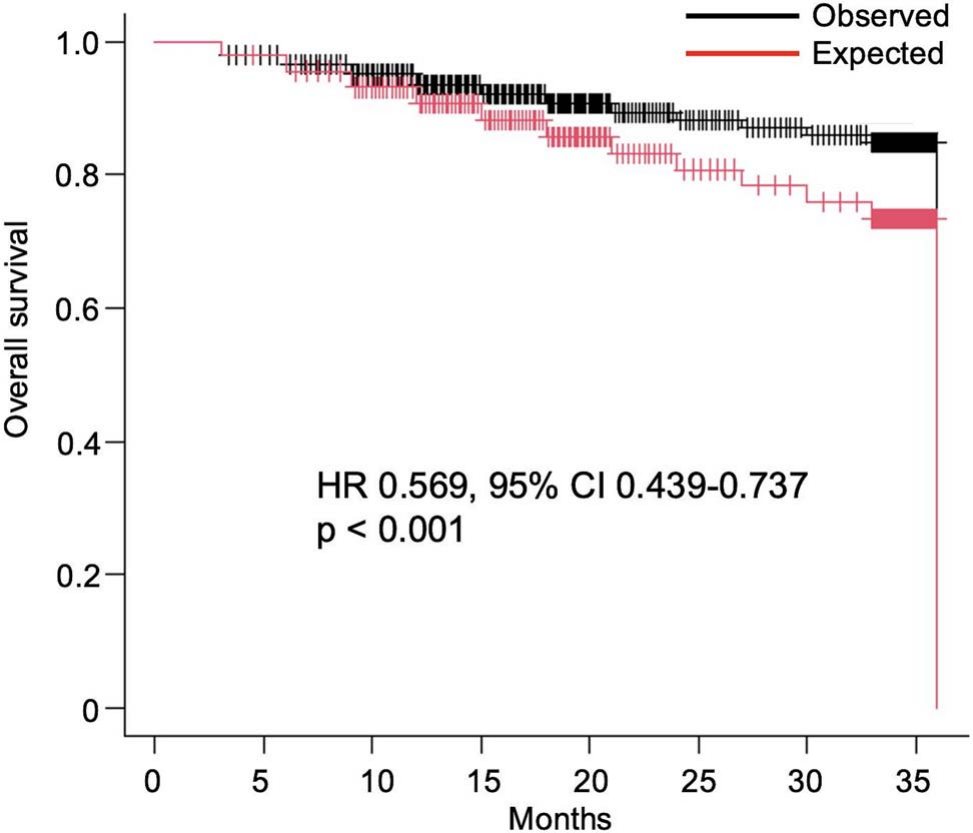
Kaplan–Meier curves of observed versus predicted time to initial subsequent systemic anticancer therapy. The black line represents the observed time to initial subsequent systemic anticancer therapy (TSSAT) of the triplet regimen, based on reconstructed individual patient data (IPD) from the ARASENS trial. The red line represents the predicted TSSAT, generated by an independent drug action model using data from both the ARASENS and ARANOTE trials. The hazard ratio (HR), 95% confidence interval (CI), and *P*-value shown were derived from a Cox proportional hazards model comparing the two reconstructed cohorts.

The Cox proportional hazards model confirmed this finding, showing that the observed triplet therapy was associated with a significant reduction in the risk of death compared to the predicted outcome (HR 0.824, 95% CI 0.681–0.998; *P* = .047). Furthermore, the supplementary analysis using TSSAT as an endpoint yielded consistent findings; the observed TSSAT was significantly longer than predicted (HR 0.569, 95% CI 0.439–0.737; *P* < .001) (Fig. 2). These results indicate a modest but statistically significant clinical benefit beyond the expected additive effect for both OS and TSSAT, providing quantitative evidence that supports a greater-than-additive interaction.

## Discussion

This study provides the first quantitative, clinical data-driven evidence suggesting that a synergistic interaction contributes to the OS benefit of the ARASENS triplet regimen in mCSPC. By applying a validated modeling approach, we were able to dissect the nature of the interaction between darolutamide and docetaxel, a question of significant clinical and biological importance. This methodology has been recently applied in urologic oncology to assess drug interactions in advanced urothelial cancer, demonstrating its utility in interpreting the results of combination therapy trials [10].

Our finding of a greater-than-additive effect has significant clinical implications. It provides a strong biological rationale for the upfront, simultaneous administration of these agents. The observed benefit in TSSAT, an endpoint preceding subsequent therapies, suggests that the full potential of this combination may be best captured through concurrent exposure rather than a sequential approach where one agent is reserved for later lines of therapy. Our quantitative estimate—an 18% risk reduction beyond the expected additive effect—helps clinicians and patients better understand the magnitude of the incremental benefit gained from the intensive triplet combination, supporting its use as a primary therapeutic strategy in appropriate mCSPC patients. This clinical observation is consistent with recent preclinical work by Buck *et al.*, which demonstrated that darolutamide augments docetaxel’s antitumor effect by reinforcing G1 cell-cycle arrest, providing a plausible biological mechanism for the synergy we detected [11].

Several limitations of our study warrant consideration. First, a key consideration is the influence of subsequent therapies on OS. As subsequent docetaxel use was limited in the ARANOTE control arm (28.8%) [7], the observed OS benefit could partially reflect the advantage of an upfront triplet treatment sequence over a strategy where docetaxel is deferred. However, our supplementary analysis on TSSAT, an endpoint unaffected by postprogression treatments, also demonstrated a significant benefit beyond the predicted additive effect. This suggests the presence of an early therapeutic advantage that cannot be solely attributed to differences in subsequent therapy.

Second, this analysis relies on a cross-trial comparison of aggregate data. Inherent heterogeneity between the ARASENS and ARANOTE study populations, such as the higher proportion of de novo metastatic disease in ARASENS (Supplementary Table S1) [12], represents a major source of potential bias for which we could not adjust. Finally, the statistical significance of our findings was borderline, and the use of reconstructed IPD introduces a degree of uncertainty. Therefore, our results should be considered exploratory and hypothesis-generating, supporting a potential for a greater-than-additive interaction rather than providing conclusive proof of synergy.

In conclusion, despite these limitations, our modeling analysis provides evidence that the triplet regimen of darolutamide, ADT, and docetaxel may confer a clinical benefit beyond the predicted additive effect. While this finding supports the rationale for upfront intensive therapy, the modest and borderline nature of this effect suggests that the incremental benefit may not be uniform across all patients. Rather than viewing the triplet regimen as a universal standard, our findings emphasize the importance of future research to identify patient subgroups—through clinical or genomic biomarkers—who are most likely to derive substantial benefit from this intensive combination.

## Supplementary Material

Table1_hyaf211
